# Benchmarking current preslaughter management factors, welfare indicators, and meat quality outcomes at commercial fed cattle processing facilities in the United States

**DOI:** 10.1093/tas/txad150

**Published:** 2024-01-03

**Authors:** Melissa K Davis, Paxton A Sullivan, Ann M Hess, Mahesh N Nair, Daniel F Mooney, Lily N Edwards-Callaway

**Affiliations:** Department of Animal Sciences, Colorado State University, Fort Collins, CO 80523, USA; Department of Animal Sciences, Colorado State University, Fort Collins, CO 80523, USA; Department of Statistics, Colorado State University, Fort Collins, CO 80523, USA; Department of Animal Sciences, Colorado State University, Fort Collins, CO 80523, USA; Department of Agricultural and Resource Economics, Colorado State University, Fort Collins, CO 80523, USA; Department of Animal Sciences, Colorado State University, Fort Collins, CO 80523, USA

**Keywords:** bruising, cattle, mobility, preslaughter, transportation, welfare

## Abstract

Preslaughter management factors and their impacts on cattle welfare and meat quality are well documented in current literature. However, certain management factors related to transportation and lairage are underrepresented. Benchmarking preslaughter management factors that can impact welfare and meat quality outcomes will allow the industry to identify areas for improvement. The objective of the current study was to benchmark preslaughter management factors for a nationwide sample of commercial fed cattle processing facilities. Five processing facilities in the West, Midwest, and Southwest regions of the United States were sampled from March 2021 to July 2022. Data were collected on a total of *n* = 637 slaughter lots representing *n* = 87,220 head of cattle. Variables of interest included general cattle characteristics, distance traveled to the plant, truck wait times to unload, environmental conditions, lairage density, and lairage duration. Additionally, mobility was scored using a four-point locomotion scale (one being normal, not lame, and four being extremely reluctant to move). Carcasses were also observed for bruising using the following scale: no bruises, bruises smaller or larger than a deck of cards, and having multiple bruises. Descriptive statistics were performed on the data at the lot and individual animal level. On average, cattle traveled 155.8 ± 209.6 km (mean ± SD) from the feedlot to the processing facility and waited 30.3 ± 39.7 min to unload. Once in lairage pens, cattle were held for 200.7 ± 195.0 min. The mean lairage stocking density was 3.1 ± 2.0 m^2^ per head. A majority of the cattle scored a mobility score of 1 (*n* = 77,645, 91.8%), 7.8% (*n* = 6,125) were scored as a two and the remaining less than one percent of cattle were scored as either a 3 or 4 (*n* = 265). Carcasses with bruises less than or equal to the size of a deck of cards (*n* = 22,672, 27.1%) were less frequent than bruises measuring greater than the size of a deck of cards (*n* = 34,427, 42.6%). Of carcasses that were bruised, 65.2% (*n = *39,856) had multiple bruises of varying size. This baseline data on preslaughter management factors identifies opportunities for improvement in wait times, lairage densities, and factors that cause bruising. Future studies should explore the relationships between these factors and their impacts on welfare and meat quality, report the economic value of these outcomes, and explore industry acceptability and adoptability of optimal preslaughter management practices.

## Introduction

As a complex and critical time in the beef supply chain, the preslaughter phase (beginning when animals are loaded for transport at the feedlot and ending with stunning and slaughter) represents a period in which cattle may encounter multiple stressors. In the United States, federal regulations (9 CFR Part 313; United States Electronic Code of Federal Regulations, 2023) require that livestock be handled with “minimum excitement and discomfort”, and this is applicable to all preslaughter processes. Most processing facilities in the United States also adhere to the recommended animal handling guidelines developed by the North American Meat Institute (NAMI, 2021) to promote and monitor animal welfare within their facilities. These guidelines also provide thresholds of acceptability for several preslaughter management factors (e.g., water provision in lairage, or truck waiting time to unload animals at the plant). Additionally, consumer pressure for high standards of animal welfare at slaughter facilities (Edwards-Callaway and Calvo-Lorenzo, 2020) is undoubtably a major factor in the global push for additional focus on animal welfare standards and requirements during the preslaughter period (Terlouw et al., 2008; Choe, 2018).

As cattle are exposed to the multiple stressors during this preslaughter phase (e.g., transportation, commingling, novel environments, and interactions with unfamiliar humans) they exhibit physiological and behavioral changes as they attempt to cope with the new environment. The stress induced by these challenges can impact both cattle welfare and meat quality. There are many indicators that can be used to assess cattle welfare during the preslaughter period (Davis et al., 2022b). Cattle mobility has garnered increased focus as an important and relevant welfare metric for fed cattle arriving at slaughter facilities (Losada-Espinosa et al., 2018; Edwards-Callaway and Calvo-Lorenzo, 2020; Mijares et al., 2021), yet mobility as a welfare indicator was not as prevalent in the research included in the Davis et al. (2022b) scoping review. However, cattle mobility has been included in other national benchmarking efforts (e.g., Elanco’s Cattle Mobility Assessment Program), and recently added to the National Beef Quality Audit (NBQA; Eastwood et al., 2017) underlining its importance. Additionally, the prevalence of carcass bruising and dark cutting (DC) carcasses across studies is highly variable, yet well researched (Sullivan et al., 2022), and offer insight as significant indicators of welfare. These welfare indicators, among others, serve as useful tools to assess and monitor animal welfare in real-time.

While great progress has been made in the U. S. cattle industry to ensure that stress is minimized and positive animal welfare states are promoted during this terminal point in cattle production cycle (NAMI, 2021; NCBA, 2022), it is important to continually measure how preslaughter management factors impact cattle welfare and meat quality. A growing body of research on preslaughter stress not only in the United States, but globally, articulates the strong relationship between preslaughter management factors and animal welfare and meat quality outcomes (Adzitey, 2011; Edwards-Callaway and Calvo-Lorenzo, 2020; Davis et al., 2022b; Sullivan et al., 2022). Factors related to transportation, animal handling, and lairage are consistently among the most commonly researched predictors for welfare outcomes of beef cattle at slaughter (Miranda-de la Lama et al., 2014; Davis et al., 2022b). However, waiting time at the plant to unload cattle, lairage duration, and density as influencing factors are underrepresented in current literature despite their relevance to the preslaughter period (Davis et al., 2022b; Sullivan et al., 2022).

There is little aggregate data at a national scale benchmarking some of these critical preslaughter management factors, such as time to unload and lairage density and duration. There have been some national efforts to quantify a selection of preslaughter management factors and cattle welfare and meat quality outcomes. The National Cattlemen’s Beef Association (NCBA) NBQA evaluates current transportation, mobility, and quality characteristics of U.S. cattle on a large scale (Boykin et al., 2017; Eastwood et al., 2017) yet is not animal welfare focused and thus does not offer an in-depth assessment of additional welfare indicators. The NBQAs benchmark the beef industry efforts every 5 yr to evaluate its improvements in specific aspects of beef quality. By benchmarking animal welfare throughout the preslaughter phase, this sector of the industry has the same opportunity to identify needs and make improvements. Understanding how cattle are managed during the preslaughter period can provide valuable information to help stakeholders identify potential areas for improvement in addition to demonstrating the progress that has been made in managing cattle during this critical time. Thus, the objective of this study was to benchmark fed cattle preslaughter management factors, welfare indicators, and meat quality outcomes at commercial processing facilities in the United States.

## Materials and Methods

### Ethical Statement

All animal data was collected via observation or obtained from plant records, and therefore the Colorado State University Animal Care and Use Committee granted this research exemption from IACUC oversight (IACUC Exemption #2019-080-ANSCI).

### Characteristics of Processing Facilities

Five U. S. Department of Agriculture (USDA) inspected commercial processing facilities in the West, Midwest, and Southwest regions of the United States were sampled from March 2021 to July 2022. Data were strategically collected at each facility multiple times throughout the collection process to capture variability in environmental conditions throughout the seasons. Four plants operated two 8-h production shifts per day averaging ~4,000 to 5,000 cattle slaughtered per day with chain speeds ranging from ~250 to 390 cattle per hour. One plant operated only one shift per day slaughtering ~1,200 cattle per day at a line speed of ~150 cattle per hour. In total, data were collected on *n* = 637 slaughter lots (i.e., groups of cattle originating from the feedlot that will remain a group throughout the process and given a slaughter lot identification number) representing *n* = 87,220 head of cattle. For plants with two shifts, data were collected from both shifts to capture additional variability (shift 1: *n* = 248 lots, 48.1%; shift 2: *n* = 268 lots, 51.9%). Slaughter lots of cattle were tracked, using their individual lot identification number, from arrival at the facility through the slaughter process and both antemortem and postmortem observational measurements were collected.

### Cattle Population

Sex class (steer, heifer, or mixed-sex lot), hide color (no black [i.e., all red, white, gray, etc.], all black, > 50% black, ≤ 50% black, or Holstein patterned), and general breed type (predominantly *Bos taurus*, *Bos indicus,* or Holstein) were recorded at the lot level. Cattle were considered to have *B. indicus* influence if more than 25% of the cattle within the lot possessed two or more of the typical breed characteristics (e.g., large droopy ears, excess skin on their dewlap and/or prepuce, or a large hump on their withers). The population of cattle in this study was predominantly >50% black-hided steers (*n* = 391 lots, 65.9% and *n* = 337 lots, 56.4%, respectively) of *B. taurus* influence (*n* = 520 lots, 88.7%). The number of animals per lot was recorded during observation and the average live weight for each lot was obtained from plant records when available. The average live weight in this study was 623.6 ± 60.4 kg (mean ± SD).

### Transportation

The lots of cattle in this study originated from feedlots where they were loaded onto cattle trucks and transported to their respective processing plant. Feedlots of origin were recorded for each lot and Google Maps (Google LLC, Mountain View, CA, USA) was used to calculate an approximate transport distance (distance traveled) between each feedlot and the plant. Multiple routes were often suggested in Google Maps; however, the first route recommended, typically representing the shortest distance and/or travel time, was selected. Upon arrival at the plant, each truck’s arrival time was recorded and later subtracted from the time the cattle were unloaded (i.e., the time at which the first animal stepped off the truck), to calculate the amount of time cattle waited on the trucks at the plant to unload (truck waiting time). Truck waiting time for each truck within the lot was then averaged to calculate truck waiting time at the lot level. Cattle at each plant were handled and managed by plant employees according to each plant’s standard operating procedures beginning at unloading from the trucks.

### Lairage

After unloading, cattle were moved to lairage pens. While in the lairage pens at each plant, cattle were provided ad libitum access to water and during the warmer months, sprinklers were turned on at either 26.7 or 32.2 °C depending on the plant. One plant had permanent shade cloths above all pens with sprinklers underneath. Most plants had stamped concrete flooring in the handling areas and lairage pens, however, one plant had rubber-matted floors in all handling areas and lairage pens except for the unloading docks and scale. Cattle receiving times varied by plant, therefore slaughter lots of cattle were occasionally held overnight in lairage before slaughter the next day.

Prior to being slaughtered, all cattle were inspected by a USDA inspector. After antemortem inspection, cattle were moved through the drive alleys, into the single-file, and up to the center track restrainer where they were rendered insensible using pneumatic captive bolt stunners and exsanguinated before further processing. One day a week, one plant used a head restrainer for Halal slaughter on lots of Holstein and native cattle. Immediately following the neck cut made by a certified Imam, a captive bolt hand-held stunner was used to render the animal insensible.

#### Mobility and heat stress scoring.

Multiple trained mobility scorers were used throughout the study period. Training videos were used to ensure that scorers received a Kappa coefficient for inter-observer reliability ≥0.80 (i.e., strong to almost perfect agreement; McHugh, 2012) compared to a gold standard scorer to qualify as a mobility scorer. Cattle mobility was scored at one time point throughout the preslaughter process, typically when in the drive alley as cattle were moved to slaughter after resting time in lairage or when moving between lairage pens during antemortem inspection. Mobility scorers observed cattle from catwalks above the pens or drive alleys when available. A four-point mobility scoring scale (one being normal with no apparent lameness to four being extremely reluctant to move; Table 1; NAMI, 2015) was used to score cattle mobility. The number of animals in each category was tallied and then the frequency of each mobility category was calculated per lot. The mobility scorer also recorded hide color and breed type at the lot level, and assessed cattle in lairage for signs of open mouth breathing (OMB) as a measurement of heat stress using an adapted definition developed by Mader et al. (2006; i.e., increased respiration and heavy open mouth panting with saliva usually present, and may be accompanied by a protruding tongue and the neck extended forward). For each lot, the total number of cattle exhibiting signs of OMB was recorded. The number of cattle reported as dead or a downer (i.e., arrived dead, died during lairage, or could no longer stand or walk on its own and therefore was euthanized) was also recorded for each lot.

**Table 1.** North American Meat Institute (NAMI, 2015) mobility scoring system for finished cattle

Mobility score	Definition
1	Normal, walks easily, no apparent lameness, and no change in gait
2	Exhibits minor stiffness, shortness of stride, slight limp, and keeps up with normal cattle
3	Exhibits obvious stiffness, difficulty taking steps, obvious limp, obvious discomfort, and lags behind normal cattle
4	Extremely reluctant to move even when encouraged, statue like

#### Lairage duration and density.

The total time that cattle spent in pens (lairage duration) for each lot was calculated by subtracting the time at which the middle truck of the lot unloaded from the time at which the mobility scorer started mobility scoring that lot of cattle. Lairage density reported as m^2^/animal for each lot was calculated by using the total square meters of each pen and dividing by the number of cattle in that pen.

### Environmental Conditions

Temperature (°C), humidity (%), precipitation (cm), and wind speed (km/h) were recorded at three time periods during each lot’s preslaughter process (i.e., the average time of all truck arrival times at the facility, the average time of all unloading times, and the average time of all mobility scoring) using an online commercial weather service (Weather Underground, San Francisco, CA, USA). Environmental conditions were then averaged over the three time points for each lot. Temperature and humidity were then used to calculate a Temperature Humidity Index (THI) score for each lot using the equation THI = 0.8*T + RH*(T-14.4) + 46.4 where *T* is ambient or dry-bulb temperature (°C) and RH is relative humidity expressed as a proportion (LiveCorp and Meat and Livestock Australia, 2023).

### Bruising and Meat Quality

#### Bruise scoring.

Multiple trained bruise scorers were used throughout the study period. In-plant training videos and in-person viewing experiences were used for bruise-scoring training to ensure that scorers received a Kappa coefficient for inter-observer reliability of ≥0.80 compared to a gold standard scorer to qualify as a bruise scorer. Bruise scoring occurred on the slaughter line after the hide was removed and prior to carcass splitting at each plant. The bruise scoring system used in this study was adapted from the NBQA bruise scoring system (a 10-point scale visually estimating bruise size, weight, location, and severity). The NBQA’s severity categories range from minimal to extreme where the largest bruise size in the minimal category is deemed equivalent to the size of a deck of cards, therefore a deck of cards was used as the threshold in the simplified scoring system in the present study. Three mutually exclusive categories were used to assess carcass bruising in this study; individual carcasses were scored and recorded as either having no bruises, one bruise that was ≤ to the size of a deck of cards, one bruise that was > than the size of a deck of cards, and an additional category recording if there were multiple bruises was also used (the size of the largest bruise was also noted). Within each bruise category, the number of carcasses with a bruise of the specified size was tallied and then the frequency of each category was calculated per lot. This system was used to simplify the assessment of bruises in real-time at large plants with fast chain speeds for hours at a time.

#### Carcass characteristics.

After carcasses were chilled at each plant for ~24 h postmortem, all standard operating procedures for processing were performed by plant employees. After USDA employees assigned a quality grade (QG) and yield grade (YG) to each carcass, USDA grades, hot carcass weights (HCW), dressing percentages (DP), and number of DC for each lot were obtained from each plant’s records. One plant reported DC, quality, and YGs for each carcass side, therefore in some cases, the number of carcasses reported does not equal a whole number. In the event that one carcass side was condemned, then the other carcass side is still represented in the data.

### Statistical Analysis

All statistical analyses were performed in SAS 9.4 (SAS Institute, Cary, NC). Slaughter lot (*n* = 637) was used as the observational unit. For all variables of interest, descriptive statistics were summarized at the lot level. Continuous variables were summarized by their minimum, mean, maximum, and standard deviation and categorical variables by relative frequency. The relative frequencies for the categorical variables of mobility, downer or dead cattle, OMB, bruising, DC, QG, and YG were calculated as a proportion using individual animal data (*n* = 87,220). Percent within optimal range for the variables truck wait time, lairage density and THI values were also calculated in SAS 9.4 (SAS institute, Cary, NC) using industry guidelines. For truck waiting time, 60 min was used as the maximum optimal threshold as the NAMI Recommended Animal Handling Guidelines and Audit Guide states that the plant will receive full points during the audit if unloading of cattle is started within 60 min of the truck’s arrival at the plant (NAMI, 2021). For lairage density, a minimum density of 2.04 m^2^ was used as the optimal threshold as the average live weight across all lots was 623.6 kg, which was closest to the recommended density (2.04 m^2^) for a group of animals with an average live weight of 635 kg as also stated in the NAMI Recommended Animal Handling Guidelines and Audit Guide (NAMI, 2021). A THI value below 72 is considered normal with no risk of heat stress in the Australian Veterinary Handbook for Cattle, Sheep, and Goats (LiveCorp and Meat and Livestock Australia, 2023), and therefore 72 was used as the maximum threshold for optimal values for THI.

## Results

Within each company and plant, differences in preslaughter management, facility design, and data management resulted in inconsistent sample sizes across each variable as noted in the tables throughout. It is also important to consider that the results presented in this study are averages or frequencies from the entirety of the project and span all four seasons, therefore do not represent season specific challenges that may have occurred (e.g., environmental conditions).

### Cattle Population

Detailed cattle population characteristics are presented in Table 2. A total of *n* = 637 slaughter lots representing *n* = 87,220 head of cattle were sampled in this study. Sex class, breed type, and hide color were recorded for 598, 586, and 594 lots, respectively. Over half (56.4%, *n* = 337) of the lots were steers and 31.9% (*n* = 191) were heifers and the remaining 11.7% (*n* = 70) were lots of mixed sex. A majority of lots (*n* = 520, 88.7%) consisted of cattle with predominantly *B. taurus* influence and 11.3% (66) of lots consisted of either Holsteins (*n* = 21, 3.6%) or cattle that were ≥ 25% *B. indicus* influence (*n* = 45, 7.7%). Slaughter lots with no black-hided cattle were infrequent (*n* = 20, 3.4%), whereas lots of cattle with >50% black or all black cattle made up 65.9% (*n* = 391 lots) of the sample population. A little less than one-third of the lots consisted of cattle with ≤ 50% black hides (*n* = 162, 27.3%). The number of cattle per slaughter lot averaged 136.9 ± 95.1 (Mean ± SD) with a range from 1 to 929 cattle, and average live weight was 623.6 ± 60.4 kg ranging from 361.5 to 766.4 kg.

**Table 2.** Characteristics of fed cattle slaughter lots during the present study

Characteristic	*n*	Frequency, %
Sex class (*n* = 598)		
Steers	337	56.4
Heifers	191	31.9
Mix^1^	70	11.7
Breed type^2^ (*n* = 586)		
*Bos taurus* or < 25% *Bos indicus* influence	520	88.7
≥ 25% *Bos indicus* influence	45	7.7
Holstein	21	3.6
Hide color (*n* = 594)		
No black	20	3.4
All black	36	6.1
> 50% black	355	59.8
≤ 50% black	162	27.3
Holstein	21	3.5

^1^Slaughter lots consisting of both steers and heifers were considered a mix lot.

^2^Cattle were considered to have *Bos indicus* influence if more than 25% of the cattle within the lot possessed two or more of the typical breed characteristics (e.g., large, droopy ears, excess skin on their dewlap and/or prepuce, or a large hump on their withers).

### Preslaughter Management Factors

#### Transportation and lairage.

Descriptive statistics on transportation and lairage factors are presented in Table 3. Cattle in the present study originated from 148 different feedlots throughout the West, Midwest, and Southwest regions of the United States and traveled on average 155.8 ± 209.6 km (mean ± SD) to the plant, ranging from 2.7 to 1,332.5 km. Once trucks arrived at each processing facility, trucks waited on average 30.3 ± 39.7 min ranging from 0 to 574.2 min before unloading their cattle. A total of *n* = 521 (85.8%) slaughter lots of cattle were unloaded within the optimal time of 60 min.

**Table 3.** Potential risk factors during preslaughter transportation and lairage associated with animal welfare and meat quality outcomes at the slaughter lot level

Variable	*n*	Minimum	Mean	Maximum	SD	*n*,% Optimal^3^
Transportation						
Distance traveled, km	604	2.7	155.8	1,332.5	209.6	—
Truck waiting time, min	607	0.0	30.3	574.2	39.7	521, 85.8
Lairage						
Lairage duration, min	572	4.0	200.7	1,071.5	195.0	—
Lairage density, m^2^/animal	609	0.6	3.1	31.7	2.0	509, 83.6
Environmental characteristics						
Temperature humidity index (THI)^1^^,^^2^	622	18.9	60.4	81.5	13.6	449, 72.2
Precipitation^2^, cm	622	0.0	0.004	0.25	0.03	—
Wind speed^2^, km/h	622	0.0	18.1	56.3	10.1	—

^1^THI score was calculated using the equation: THI = 0.8*T + RH*(T-14.4) + 46.4 where T is ambient or dry-bulb temperature (°C) and RH is relative humidity expressed as a proportion (LiveCorp and Meat and Livestock Australia, 2023).

^2^Temperature and humidity used to calculate THI, precipitation, and wind speed were recorded using an online commercial weather service (Weather Underground, San Francisco, CA, USA).

^3^Percent within optimal range is based on thresholds stated in the NAMI Recommended Animal Handling Guidelines and Audit Guide (NAMI, 2021) and the Australian Veterinary Handbook for Cattle, Sheep, and Goats (LiveCorp and Meat and Livestock Australia, 2023). The threshold used for truck waiting time was 60 min or less, the threshold used for lairage density was 2.04 m^2^ or above, and the threshold for THI value used was 72 or below.

In lairage, slaughter lots spent on average 200.7 ± 195.0 min waiting before slaughter, and the durations ranged from 4 to 1,071.5 min. Lairage density, or sometimes referred to as stocking density, was 3.0 ± 2.0 m^2^/animal with a range of 0.6 to 31.7 m^2^/animal, and *n* = 509 (83.6%) slaughter lots of cattle experienced lairage densities within the threshold minimum of 2.04 m^2^/animal based on the average liveweight of the population.

#### Environmental characteristics.

THI, precipitation, and windspeed descriptive statistics are reported in Table 3. Temperature and relative humidity were used to calculate a THI score for each lot which averaged 60.4 ± 13.6 and ranged from 18.9 to 81.5 over all lots in the current study. A total of *n* = 449 (72.2%) of cattle slaughter lots experienced weather conditions with THI values below the optimal maximum threshold of 72. The occurrence of precipitation during data collection dates was low (0.004 ± 0.03 cm) throughout the study, however, wind speed varied from 0 to 56.3 km/h and averaged 18.1 ± 10.1 km/h.

### Preslaughter Management Outcomes

#### Mobility and open-mouth breathing.

All preslaughter management outcomes reported as a frequency are presented in Table 4. Cattle with a mobility score of 1, or no mobility impairment, was the most common amongst the cattle within the study population (*n* = 77,645, 91.8%), whereas cattle scoring a 2, or exhibiting minor stiffness or a slight limp, were less frequent (*n* = 6,125, 7.8%). Cattle with a mobility score of a 3 or 4 (i.e., extreme stiffness or reluctancy to move) equated to <0.4% (*n* = 265) of the study population. Dead or downer cattle were rarely observed in the current study (*n* = 17, 0.03%), as well as signs of OMB (*n* = 41, 0.06%).

**Table 4.** Cattle welfare and meat quality outcome variables associated with preslaughter management risk factors

Variable	Lots, *n*	*n* animals/carcasses	Frequency, %
Mobility scores^1^	614		
1		77,645	91.8
2		6,125	7.8
3		264	0.3
4		1	0.002
Downer or dead cattle	614	17	0.03
Open mouth breathing^2^ (OMB)	614	41	0.06
Bruise scores^3^	612		
None		24,858	30.3
≤ Deck of cards		22,672	27.1
> Deck of cards		34,427	42.6
Multiple^4^		39.856	65.2
Dark cutting^5^	374	873	1.6
Quality grades^5^	618		
Prime		6,888	8.6
Choice		59,338	70.6
Select		16,104	19.0
Standard		475	0.5
Other		1,162.5	1.3
Yield grades^5^	618		
1		5,169	6.4
2		28,946	33.0
3		37,121	42.3
4		11,947	14.3
5		2,879	4.0

^1^Mobility scores are defined as: 1, normal, walks easily, no apparent lameness; 2, exhibits minor stiffness, shortness of stride, slight limp, keeps up with normal cattle; 3, exhibits obvious stiffness, difficulty taking steps, obvious limp, obvious discomfort, lags behind normal cattle; and 4, extremely reluctant to move even when encouraged by a handler, statue-like (NAMI, 2015).

^2^Open mouth breathing (OMB) was assessed by recording the number of cattle in each lot exhibiting signs of OMB. A definition adapted from Mader et al. (2006; increased respiration and heavy open mouth panting with saliva usually present and may be accompanied by a protruding tongue and the neck extended forward) was used.

^3^Individual carcasses were scored and recorded as either having no bruises (none), one bruise that was ≤ to the size of a deck of cards, one bruise that was > than the size of a deck of cards, and if it had multiple bruises where the size of the largest bruise was noted. Scores were then summarized at the lot level.

^4^Multiple is expressed as the proportion of carcasses scored as either ≤ a deck of cards or > a deck of cards that were also scored as having multiple bruises.

^5^One plant reported dark cutting, quality, and yield grades for each carcass side. In the event that one carcass side was condemned, then the other carcass side is still represented in the data.

#### Bruising and carcass characteristics.

Carcasses exhibiting bruising, regardless of size, occurred more frequently (*n* = 57,099, 69.7%) than carcasses with no apparent bruising (*n* = 24,858, 30.3%; Table 4). Of carcasses that were bruised, approximately two-thirds (*n* = 39,856, 65.2%) exhibited multiple bruises. Data on the number of DC carcasses within each lot were acquired for 374 of the lots in this study due to differences in company records. Out of those lots, DC occurred in 1.6% (*n* = 873) of carcasses. More than three-quarters of carcasses in the present study received a QG of Choice or higher (*n* = 66,226, 79.2%), 19.0% (*n = *16,104) and 0.5% (*n* = 475) of carcasses received a QG of Select or Standard, respectively, and the remaining less than 2% (*n = *1,162.5) of carcasses were placed in the “Other” category (i.e., carcasses were graded as Cutter, Canter, No Roll, etc.). The YG of individual carcasses varied from 1 to 5, with a majority of the carcasses grading as either a 2, 3, or 4 (*n* = 78,014, 89.6%), and 6.4% (*n *=* *5,169) and 4.0% (*n *= 2,879) grading as a 1 or 5, respectively. HCW ranged from 301.6 to 513.8 kg and averaged 396.4 ± 36.9 kg, and DP ranged from 57.9% to 67.7% and averaged 63.6% ± 1.5% (Table 5).

**Table 5.** Cattle meat quality outcome variables associated with preslaughter management risk factors

Variable	*n*	Minimum	Mean	Maximum	SD
Hot carcass weight, kg	611	301.6	396.4	513.8	36.9
Dressing percentage, %	610	57.9	63.6	67.7	1.5

## Discussion

Benchmarking this breadth of preslaughter management factors, welfare indicators, and meat quality outcomes at this scale, in real-time, has not previously been done or reported for fed cattle in the United States. Although the number of cattle in this study represents a small subset of the number of all cattle commercially processed in the United States each year (0.24% of the ~34 million cattle slaughtered in 2022; USDA-NASS, 2023a), the data set still remains representative of the characteristics of fed cattle slaughter in the United States. For example, several variables in the present study reflect current trends in the industry (e.g., breed types and sex class proportions, cattle mobility, and carcass characteristics) and have been reported on a larger scale formerly (i.e., the NBQA; Boykin et al., 2017; Eastwood et al., 2017) with similar cattle population characteristics. Many of the variables in this study, however, (e.g., truck waiting time to unload cattle, lairage duration, and density) have never been benchmarked at this scale previously. Additionally, Texas, Nebraska, Kansas, Oklahoma, and California currently represent the top five states for cattle inventory (USDA-NASS, 2023b), all of which are located in the same regions where the plants and feedlots of cattle origin were located in this study.

### Preslaughter Management Factors

In terms of cattle characteristics, steers continue to make up the majority of cattle slaughtered in federally inspected facilities in the United States, and the frequency reported in the present study similarly reflects that. Steers represented 56.4% (*n* = 337 lots) of cattle slaughtered in the present study, similar to the 2022 USDA slaughter statistics which comprised of 47.0% steers (USDA-NASS, 2023a). Heifers represented 31.9% (*n* = 191) of cattle in the present study, similar to the 2022 livestock slaughter summary reported number of 30.6% (USDA-NASS, 2023a). The values in the livestock report, however, are based on individual animal numbers and not slaughter lots as represented in the current study; in the current study, lots of mixed sex were also recorded and could be one reason why reported frequencies of each sex class differ. Regional and company differences may also contribute to variation in sex class percentages slaughtered.

Greater emphasis has been placed on breed type and primary hide color for several years within the industry (e.g., several certified beef programs promote beef coming from Angus-influenced or black-hided cattle; USDA-AMS, 2023). In the current study, the majority of lots (*n* = 391, 65.9%) consisted of >50% or all black-hided cattle; in line with the 2016 NBQA data (57.8% black hided; Eastwood et al., 2017). The percentage of Holstein lots in this study, although, was less than what was reported in the last NBQA (3.5%, *n* = 21 vs. 20.4%, respectively; Eastwood et al., 2017). However, a recent similar study reported a slightly less percentage of Holstein cattle lot slaughtered (16%; Lee et al., 2017), suggesting that these differences in hide color and breed type, similar to differences in sex class, are likely due to differences between regions and plant cattle procurement. The *B. taurus* breed type currently dominates the U.S. cattle industry; past estimates suggest that *B. indicus*-influenced cattle typically only represent ~8% of the total U.S. cowherd (Cundiff et al., 2012); similar to the current study findings of 7.7% (*n *= 45 lots). *Bos indicus* cattle tend to have a shorter, smoother hair coat allowing them to withstand and survive in higher temperatures and humidity (Forbes et al., 1998), and therefore were typically only recorded in data collected from plants in the Southwest region of the United States.

Transportation is a highly researched, reviewed, and regulated component of the preslaughter period, not only for beef cattle, but for all livestock species (FAO, 2001; Davis et al., 2022b; Hultgren et al., 2022). Transportation represents one of the most stressful events that cattle may experience in their lifetime even in favorable conditions (Kettlewell and Mitchell, 2005; Schuetze et al., 2017), and some cattle may experience transportation for the first time when transported to slaughter depending on their origin or production system. In this study, a wide range of transportation distances were recorded (2.7 to 1,332.5 km), consistent with transportation distances reported in the last NBQA (12.9 to 1,400.1 km; Eastwood et al., 2017). While there are currently no regulations regarding transportation distances in the United States, a federal regulation exists restricting the amount of time livestock can be transported without rest, feed, and water. As its name suggests, the Twenty-Eight Hour Law (1994) mandates that cattle cannot be transported longer than 28 h; however, this law is not strictly enforced and is difficult to do so. Although not directly measured in this study, based on the maximum distances traveled, cattle in this study likely were not transported for more than 28 h. There are a number of factors that could influence where cattle are shipped for slaughter including but not limited to company procurement agreements, location of a plant in the area, cattle type, and scheduling needs. Transportation distances as reported in this study and do not necessarily accurately estimate transportation duration as distance alone does not capture delays during transport or road conditions, all of which may in some capacity have an impact on animal welfare and meat quality (Schwartzkopf-Genswein et al., 2012; Miranda-de la Lama et al., 2014).

To the best of the authors’ knowledge, truck wait times to unload cattle at slaughter facilities have not been benchmarked or researched previously despite the existence of current acceptable thresholds for truck wait time in industry audit guides; the NAMI Recommended Animal Handling Guidelines and Audit Guide (NAMI, 2021) requires 60 min or less wait time to unload in order to receive full points on this audit criterion. A total of *n* = 521 (85.8%) of slaughter lots in this study were within this optimal threshold underlining an important success in this sector of the industry. Many processing plants have policies in place to minimize wait times at the plant by implementing a scheduling system to coincide with the slaughter schedule and holding pen capacity, nevertheless, delays and various unforeseen events can result in a backlog of trucks waiting to unload. In the current study, the average truck waiting time was ~30 min, however, the wide range of wait times, with a maximum of 574 min (~9 and a half hours), is a concern. Past studies have reported that extended time on the truck increases risks of injury, fatigue, or potentially death from the increased standing time and/or weather-related stress, especially when cattle are without adequate airflow (Minka and Ayo, 2007; Goldhawk, 2014; Schuetze et al., 2017; NAMI, 2021). Processing companies often have protocols in place that require management personnel to instruct truck drivers to continue driving when wait times to unload at the plant are long and temperatures exceed the company’s maximum heat threshold (e.g., 27 °C; personal communication L.N. Edwards-Callaway); however, many drivers do not leave, likely due to risk of missing their opportunity to unload or not wanting to incur additional fuel costs with the added distance.

Lairage duration, or the time that animals spend resting in holding pens at the processing plant prior to being slaughtered, varies from plant to plant. Generally, during this time, antemortem inspection by the Food Safety Inspection Service inspectors occurs; antemortem inspection can take place at different times (i.e., during unloading or immediately before slaughter) depending on the facility. Lairage also allows for the entirety of a slaughter lot of animals to arrive and allows the animals time to rest following transportation (Warriss et al., 1992; Edwards-Callaway and Calvo-Lorenzo, 2020). Some plants receive cattle the evening prior to the next kill shift to rest in lairage overnight until the morning. Regardless of lairage duration, federal regulations in humane livestock slaughter require that cattle always have access to water, access to feed if held longer than 24 h, and have sufficient space to lie down when held overnight (CFR—Humane Slaughter of Livestock., 1979). Surprisingly, there is no industry benchmarking data on current lairage durations at processing plants. Few studies have researched the effects of varying lairage durations on fed cattle welfare during the preslaughter period at plants in the United States. There is currently contradicting research on the impact of lairage duration on cattle welfare and meat quality outcomes (Ferguson et al., 2007; Teke et al., 2014; Özdemir et al., 2022). For example, Liotta et al. (2007) found that longer lairage durations (58 h vs. 31 h) significantly increased ultimate pH values, whereas Ferguson et al. (2007) found no effect on ultimate pH for cattle held in short versus long lairage durations (3 h vs. 18 h, respectively). Lairage duration may be a difficult variable to compare across studies as perceptions of “short” vs. “long” lairage durations and conditions during lairage vary from study to study which may confound the results. In the current study, a wide range of lairage durations were recorded (4 to 1,071.5 minutes), but the average duration was ~200 min, or just over three hours. This benchmarking information could be valuable for future analyses exploring how lairage duration impacts fed beef cattle welfare and subsequent carcass quality.

Stocking densities in lairage are largely determined by the NAMI space allowance guidelines as well as by available space for incoming cattle and the need to maintain groups of cattle together without mixing (NAMI, 2021). Both the plant-to-plant and in-plant variation in lairage stocking density are highly variable. As mentioned earlier, federal regulations in humane livestock slaughter require cattle to have sufficient space to lie down when held overnight (CFR—Humane Slaughter of Livestock., 1979) but are not prescriptive in the exact space required per animal. The NAMI guidelines also have recommendations for various space allowances for cattle based on average weight; however, this parameter is not included in the audited segment of this tool (NAMI, 2021); space allowance is a secondary audit criterion and is included as essentially a note in the audit. While the average lairage density in the present study was 3.1 m^2^/animal, (well above the minimum space allowance of 2.04 m^2^/635 kg. animal in the NAMI guidelines; NAMI, 2021) the minimum reported density of 0.6 m^2^/animal is over three times less than the minimum recommendation. At this space allowance, water access and room available to lie down is limited, and there is likely increased temperature and humidity in the pen microclimate. While the average stocking density of lairage pens is well within regulations and guidelines, and 83.6% (*n* = 509) of slaughter lots were above the minimum threshold of 2.04 m^2^/animal, increased attention and focus on ensuring that all stocking densities in lairage are following federal regulations and the NAMI guidelines is critical for allowing cattle the space and opportunity to access water and lie down.

The THI provides thresholds of when heat mitigation or applying different handling methods should be implemented to help alleviate heat stress in cattle (Chichester and Mader, 2012; UNL Beef, 2014). The severity of heat stress is related to the ambient temperature and the level of humidity (Gantner et al., 2011), both of which are factored into the equation for calculating a THI value (THI = 0.8*T + RH*(T-14.4) + 46.4) where T is ambient or dry-bulb temperature (°C) and RH is relative humidity expressed as a proportion; LiveCorp and Meat and Livestock Australia, 2023). Generally, a THI value of <72 is optimal for cattle to exhibit no signs of heat stress and anything above that value can result in mild to very severe heat stress (LiveCorp and Meat and Livestock Australia, 2023). In the present study, THI values averaged 60.4, and 72.19% (*n* = 449) of cattle slaughter lots experienced weather conditions below the threshold of a THI value of 72 for heat stress symptoms to occur in cattle. However, a maximum value of 81.5 was reached at one point during the duration of the study which is within the range of causing severe stress to cattle (LiveCorp and Meat and Livestock Australia, 2023).

Commercial beef slaughter plants across the U.S. have adopted several widely used forms of heat mitigation for cattle in lairage pens. Sprinklers or misters and shade are the most widely used forms of heat mitigations; however, several other techniques such as the use of fans or the use of one technique in conjunction with another are common factors among slaughter facilities (Davis et al., 2022a). Heat mitigation via sprinklers allow for soaking of cattle hair coats to completely wet the cow, while fans facilitate air movement and increase convection to decrease respiration rates and overall body temperatures (Armstrong, 1994; West, 2003; Polsky and von Keyserlingk, 2017). Precipitation throughout data collection was low, however, averaging 0.004 cm. Conversely, increased wind speeds were recorded more frequently with an average of 18.1 km/h and a maximum speed of 56.3 km/h at one time point, both of which surpass the recommended air circulation speeds from 3.5 to 5 mph (5.6 to 8.0 km/h) to increase convective heat transfer and enhance evaporation in resting, feeding and holding areas for cattle (McFarland, 2022). However, windspeeds and precipitation in this study represent an average across all plants throughout all seasons, and therefore some plants may have experienced little to no wind at all. Additionally, low prevalence of precipitation reported in this study furthers the need for added, maintained, and managed sprinkler systems in lairage pens to combat added heat stress or used as preventative measures in situations where temperatures exceed the threshold for cattle comfort.

### Preslaughter Management Outcomes

OMB or a high respiration rate, typically seen as a response to heat stress, has been researched extensively in feedlot and dairy settings (Mader et al., 2006; Hagenmaier et al., 2016; Unruh et al., 2017; Ruban et al., 2020); however, is not commonly measured in lairage during the preslaughter phase for beef cattle and therefore offers an opportunity to be considered in future studies. Davis et al., (2022b) identified only one study researching OMB during lairage and these authors reported no cattle exhibiting signs of OMB during lairage (Hagenmaier et al., 2017). Similarly, in the current study, only 0.06% (*n* = 41) of the cattle exhibited OMB. OMB may not be readily observed when animals are being actively handled or when they are not settled (i.e., moving around) during lairage. The authors of this present study suggest exploring other methods to capture OMB either during a time when most animals in a holding pen are resting or using video recording, so that observers are not present.

The beef industry in recent years has pursued an increased focus on the mobility of finished cattle as it is not only a welfare concern, but can impact overall efficiency (Edwards-Callaway et al., 2017). Elanco’s Cattle Mobility Assessment Program database dates back to 2016 with historical data representing millions of cattle to serve as mobility benchmarking information for the industry. Additionally, the NBQA has recently added mobility scoring to their most recently published (2016) and future quality audits (Eastwood et al., 2017) highlighting the importance the industry places on monitoring mobility. Mobility issues in finished cattle are multifactorial and could be impacted by sex, body weight, days on feed, increased THI, handling practices, particularly during loading and unloading for transport, and transportation practices or conditions (Gonzalez et al., 2012; Edwards-Callaway et al., 2017; Lee et al., 2018; Mijares et al., 2021). Just over 91% (*n* = 77,645) of cattle in the present study showed no signs of impaired mobility, a small decrease from the previous NBQA’s 96.8% (Eastwood et al., 2017). On the contrary, Mijares et al. (2021) reported considerably lower frequency of normal mobility (74.6%). However, the Mijares et al. (2021) study was conducted during the COVID-19 pandemic recovery with the intention of characterizing the impacts that some of the repercussion of the pandemic had on this sector of the supply chain. The mobility scoring system used in the Elanco Cattle Mobility Assessment Program, the NBQAs, and by Mijares et al. (2021) was the same as the one used in the current study; however, the location where cattle were scored differed. For example, mobility scores in the current study were recorded as cattle were either moved between holding pens during antemortem inspection at the plant, or as they were moved into the drive alley just prior to slaughter, and therefore were not scored immediately following transportation such as in the NBQA. In the current study, only one animal (0.002%, *n* = 1) had severely impaired mobility (i.e., a score of 4). Generally, an animal that scores a 4 is immobile and will be euthanized as it will not be capable of moving through the handling facility to be slaughtered. The percentage of dead and downer (or non-ambulatory) cattle was low (0.03%, *n* = 17), similar to previously published data from other cattle transportation studies (e.g., 0.022% reported as non-ambulatory and 0.011% dying during transportation; [Gonzalez et al., 2012]). Continuing to improve efforts with animal handling, transportation, and identification of animals unfit for transport can further progress animal welfare and comfort in this sector, decrease the overall number of dead or downer cattle, improve mobility of cattle upon arrival at the slaughter facility, and allow for efficiency at the plant.

Carcass bruising as a postmortem welfare outcome serves as an indicator of a variety of potential issues that may have occurred during the preslaughter period, including inappropriate handling, rough transportation, or the presence of cattle with horns (Kenny and Tarrant, 1987; Jarvis et al., 1995; Warriss et al., 1995; Hoffman et al., 1998; Huertas et al., 2010; Strappini et al., 2010; Mendonça et al., 2019). Economic loss due to carcass bruising is also a major concern as an estimated $35 million is lost annually in the U.S. from trimming or cutting out bruises on the slaughter line that result in carcass downgrades (Gallo et al., 1999; Lee et al., 2017). The 2016 NBQA reported that ~38.9% of fed steer and heifer carcasses had at least one bruise (Eastwood et al., 2017), whereas 69.7% (*n* = 57,099) of carcasses in the present study were scored as having at least one bruise. The bruise scoring system in the present study was based strictly on size (i.e., length of the bruise), whereas the NBQA uses a scoring system based on increasing size for the first three categories and then increasing weight for the remaining seven categories. The differences between the bruise scoring systems used could have contributed to differences in reported bruising prevalence between studies. For instance, when data collectors are required to make finer discriminations (i.e., choose between more options such as in the 10-point bruise scoring scale used in the NBQA) reliability can be much more difficult to obtain (McHugh, 2012). The prevalence of bruising is also highly dependent on geographic location as well. Studies in Chile, the United Kingdom, and Uruguay have reported a wide range of carcass bruising prevalence (12.3%, 97%, and 60%, respectively; Jarvis et al., 1995; Strappini et al., 2010; Huertas et al., 2015). The prevalence of carcass bruising is increasing in recent years (Eastwood et al., 2017), and further identification and management of factors causing bruising is pertinent to the reduction of this welfare and quality issue.

Beef classified as DC is characterized by a darker color and is viewed as unfavorable to consumers (Ponnampalam et al., 2017). This occurs when cattle experience high metabolic demand (e.g., from preslaughter stress) and depletion of muscle glycogen stores, which limits the postmortem pH decline, resulting in a higher ultimate muscle pH and a darker, purplish-red muscle color (Lister, 1988). Reducing DC carcasses serves as an economic benefit as DC is estimated to cost the United States beef industry nearly $170 million annually (Underwood et al., 2007). The percentage of DC carcasses in the present study (1.6%, *n* = 873) is comparable to the 1.9% reported in the previous NBQA (Eastwood et al., 2017). Globally, the prevalence of DC reported across studies is variable and greater than that reported in the current study with studies from Mexico and Australia reporting DC prevalence of 2.8% (Pérez-Linares et al., 2015) and 39% (Steel et al., 2021), respectively. The impacts on DC beef are multifactorial and vary based on characteristics of the animal, production system, management practices, environmental factors, location, or the various identified thresholds for classifying DC throughout the literature (Sullivan et al., 2022).

Additional carcass characteristics such as YG and HCW reported in this present benchmarking study are in line with industry averages reported in the previous NBQA (Boykin et al., 2017). Additionally, an average DP of 63.6% across all lots is similar to the average DP of 63% for beef in the United States (Campbell, 2022). The majority of the carcasses in the present study were categorized as Choice (70.6%, *n* = 59,338), highly similar to the previous NBQA’s findings of 68.8% (Boykin et al., 2017). QG has the potential to be affected by preslaughter stress as lean color is a factor considered in an overall QG, and as mentioned earlier with DC, lean muscle color can potentially be affected by preslaughter stress.

## Conclusions

With the continued focus, research, and auditing of beef cattle during the preslaughter period, the industry has continued to advance and promote animal welfare focused practices. In an effort to report industry progress and identify areas needing further attention, the objective of this study was to benchmark preslaughter management factors, welfare indicators, and meat quality outcomes for fed beef cattle at five commercial processing facilities in the United States. In the present data, average truck waiting times to unload were below the recommended 60 min, and average lairage densities were well above the minimum recommendations outlined in the aforementioned NAMI guidelines (NAMI, 2021). Moreover, more than 90% of cattle had normal mobility. While it is important to highlight successes in this sector of the beef industry, it is also necessary to identify areas needing further attention. Maximum truck waiting times to unload at the plant, lairage density minimums, and the percentage of bruised carcasses, all of which are multifactorial in nature will require more research and technological advancements to overcome. More specifically, route optimization for cattle transport, clear communication between processing plants, feedlots, and truck drivers for timely delivery of cattle to the plant, and additional lairage space to unload cattle when in-plant delays occur can cumulatively minimize the amount of time cattle are on trucks. Further exploration of the impacts of truck wait time on welfare indicators and meat quality attributes of fed beef is needed to determine an appropriate threshold of optimal truck waiting time. Monitoring animal handling and evaluating cattle behavior in lairage may improve the prevalence of carcass bruising. In summary, benchmarking preslaughter management factors and subsequent welfare and meat quality outcomes for fed beef cattle in the United States is crucial to determine if the industry is meeting the standards and regulations set by governing bodies, identify areas where improvements have been made, and identify specific areas that may require further research and attention. Furthermore, this dataset will be used to investigate relationships between management factors and welfare and meat quality outcomes, report the economic value of specific welfare outcomes, and ultimately survey industry acceptability and adoptability of identified optimal management factors to improve welfare and meat quality within this sector of the beef supply chain.
